# The Effect of Smoking on Mast Cells Density and Angiogenesis in Chronic Periodontitis

**Published:** 2017-10-01

**Authors:** Noushin Jalayer Naderi, Hasan Semyari, Reza Hemmati

**Affiliations:** 1 *Dept. of Oral and Maxillofacial Pathology, Faculty of Dentistry, Shahed University*; 2 *Dept. of Periodontic, Faculty of Dentistry, Shahed University*; 3 *Faculty of Dentistry, Shahed University*

**Keywords:** Angiogenesis, Chronic periodontitis, Mast cell, Smoking, Vascular Endothelial Growth Factor (VEGF)

## Abstract

**Background and objective:**

Gingival bleeding reduction in smokers has been associated with decreased blood vessel density. The mechanism of suppressive effect of cigarette smoking on blood vessel density is not precisely defined. The aim of this study was to evaluate the impact of smoking on angiogenesis by assessing mast cells density and VEGF expression in chronic periodontitis.

**Materials& Methods:**

52 paraffin embedded block of gingiva tissues with periodontitis obtained from 30 nonsmokers and 22 smokers undergoing flap surgery were examined immunohistochemically for VEGF expression. Mast cell counts was completed on toluidine blue stained slides. Exposure to cigarette smoking was calculated by the number of packs × year. Patients were classified into 4 groups based on the number of smoked cigarettes. The correlation between VEGF expression and mast cell counts was evaluated and compared in nonsmokers and smokers.

**Results:**

The mean number of mast cells (p=0.004) and average value of VEGF expression (p = 0.000) in nonsmokers was significantly higher than smokers. No correlation was noted between VEGF expression / mast cell counts and number of smoked cigarettes in four groups of smokers (p=0.29,0.12 , 0.20 and 0.11, respectively).

**Conclusion:**

Mast cells and VEGF expression may account for suppressive effect of cigarette smoking on blood vessels in periodontitis.

## Introduction

Periodontitis is an inflammatory disease affecting teeth supportive tissue. Gingival bleeding and gingival recession, deep pockets and finally loosening of teeth are common signs of periodontitis. 

The adverse effect of cigarette smoking on periodontal health status is well established. The pocket depth and alveolar bone loss are more severe in smokers than nonsmokers (1-2).

Gingival inflammation in response to plaque collection is less in smokers than nonsmokers. Decreased gingival inflammation demonstrates by reduced gingival bleeding on probing in smokers. This suppressive effect is dose-dependent (3-4).

Reduced gingival bleeding in smokers has been associated with decreased blood vessel density and inflammatory cells infiltration in gingiva (5-6).

The mechanism of suppressive effect of cigarette smoking on blood vessel density is not precisely defined. 

The role of mast cells on acquired and innate immunity is known. In inflammatory conditions, mast cells are activated by cytokines and release pro-inflammatory mediators.

Mast cells are potent source of producing angiogenic related factors. These products have a regulating role in angiogenesis and vascular functions (7-8).

The mechanism by which mast cell mediated angiogenesis has been attributed to synthetizing pro-angiogenic micro-molecules including heparin, histamine, basic fibroblast growth factor (bFGF), vascular endothelial growth factor (VEGF) and various cytokines such as tumor necrosis factor-a (TNF-a) and interleukin (IL)-8 (9-11).

VEGF has highly mitogenic activity on endothelial cells. The role of VEGF on vascular permeability and vasoactive molecules has also been reported (12).

It has been shown that cigarette smoking stimulates chemokines in mast cells and upregulates vascular endothelial growth factor in lung tissue of pulmonary disease (13-14).

The role of smoking on blood vessel density of cigarette smoking on periodontal inflammation has not been elucidated. The aim of this study was to evaluate the impact of smoking on angiogenesis by assessing mast cells density and VEGF expression in chronic periodontitis.

## Material and Methods

This was a case-control study based on target sampling method. The study was approved by the ethical committee of Shahed University (registration number IR.Shahed.rec.1393.70). The number of samples was determined according to previous findings and using Minitab software with 95% confidence level and 90% strength of a test.

Fifty-two (30 nonsmokers and 22 smokers, mean age of 38.23 and 42.09 years. respectively) 25-50 years old, male patients with chronic periodontitis entered the study. All patients were male to remove the effect of hormones as a bias factor. The study participants were cases of gingiva flap surgery and did not impose any other intervention to them rather than their own flap surgery. 

The study participants were selected from Periodontics Dept., Faculty of Dentistry, Shahed University, Tehran, Iran, from 2013 to 2015. The inclusion criteria for both cigarette smokers and nonsmokers were presence of 20 or more teeth in mouth, not receiving periodontal treatment in the previous 6 months except for scaling and root planning and absence of systemic diseases or taking medications in medical history (6).

The exclusion criteria in nonsmokers and smokers were existing history of smoking or quitting the smoking in past three years and smoking for less than three years (15-16).

In both smokers and nonsmokers, presence of gingivitis not responding to conservative treatment with pocket formation were considered as chronic periodontitis. The gingival index (GI) and periodontal index (PI) were graded as follows; 

GI: 0= normal, 1= mild inflammation (no bleeding on probing), 2= moderate inflammation (redness, edema, and bleeding on probing), 3= severe inflammation (edema, ulceration and tendency to spontaneous bleeding)

PI: 0=negative, 1=mild gingivitis, inflammation in free gingival 2 = gingivitis, Inflammation completely circumscribes the teeth, 3=gingivitis with pocket formation. Probing pocket depth of ≥5 mm was considered as probing depth and clinical attachment loss (5).

Exposure to cigarette smoking was calculated by the number of packs × year (16). 

Based on the number of packs × year, patients were classified into 4 groups;

Group 1: from 1 to 100 packs × year

Group 2: from 101 to 200 packs × year

Group 3: from 201 to 300 packs × year

Group 4: from 301 to 400 and more packs × year

After flap surgery, the obtained specimens were fixed in 10% buffered formalin solution and embedded in paraffin. Two tissue sections were cut from each paraffin block for tissue staining.

*Evaluation of mast cells density*

The routine toluidine blue staining method was used for mast cells staining. 5 μm thick sections were cut and stained with toluidine blue after deparaffinization and rehydration. Mast cells were considered positive when demonstrated positively stained blue cytoplasmic granules.

The number of mast cells in 3 microscopic fields with higher microvascular area at magnification of 1000 × (10 × ocular and 100 × objective lenses) were counted. Mast cell density was determined as mean number/per optical field (17). Counting was double-blind and completed by optical microscopy (Zeiss, Japan).

*Evaluation of VEGF expression*

VEGF expression was detected immunohistochemically. The 3 μm sections were cut. According to the manufacturer’s instructions, sections were deparaffinized in xylene, placed in 0.01 M Citrate/HCl Buffer (pH = 6.00) and heated in microwave oven for 10 minutes. Tissue sections were rinsed with phosphate buffered saline (PBS) in room temperature and incubated with 1 μg/mL diluted primary antimouse polyclonal antibodies (Biocare, the USA) for one hour and with biotinylated antibody for 30 minutes. For imaging VEGF expression, sections were incubated in peroxidase for 30 minutes, developed in 3,3’diaminobenzidine hydrochloride (DAB) and finally Mayer’s staining. After immersing in xylene, sections were mounted. Samples were rinsed with PBS between each incubation. The phaeochromocytoma was positive control. 

**Figure 1:**
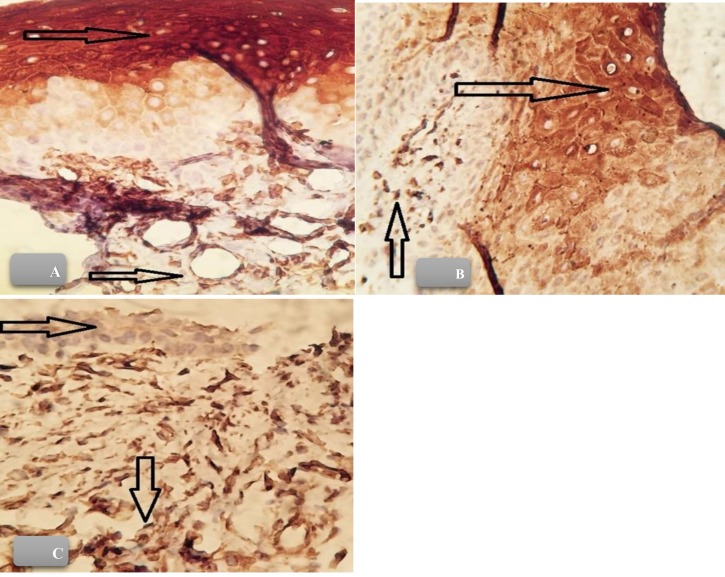
VEGF expression (100× Immunohistochemical staining). VEGF expression was graded by staining intensity of endothelial cells compared to epithelium. The brown cytoplasmic staining of epithelium was considered as positive VEGF expression. Grade 1 (A), Grade 2 (B) and Grade 3 (C) (400×).

VEGF expression was determined by intensity of staining and graded as follows (Fig.1) (18);

Grade 0: no VEGF expression

Grade +1: weaker staining intensity of endothelial cells than epithelium

Grade +2: similar staining intensity of epithelium and endothelial cells

Grade +3: stronger staining intensity of endothelial cells than epithelium.

Grading was completed double-blind. The degree of VEGF expression was determined using optical microscopy (Zeiss, Japan) in 3 microscopic fields at magnification of 100 × (10 × ocular and 10 ×objective lenses).

*Statistical analysis*

The statistically significant difference in mast cells density and VEGF expression between smokers and nonsmokers was tested using Mann-Whitney test at the level of p < 0.05. The statistically significant difference in GI and PI was tested by Mann-Whitney and ANOVA tests. The Duncan statistical test was used to analyze the difference between four smoker groups. Correlation between mast cell density and VEGF expression was tested by Pearson correlation coefficient test. Data analysis was performed by statistical software package SPSS (Version 22; Chicago, IL, USA). 

## Results

***Mast cells density***

The mean number of mast cells in nonsmokers and smokers were 9.26±2.95 and 4.81±2.95, respectively. The mean number of mast cells in nonsmokers was significantly higher than smokers (p=0.004) (Table 1).

*VEGF expression*

The average value of VEGF expression in nonsmokers and smokers were 2.33±0.88 and 1.22±0.68, respectively. The average value of VEGF expression in nonsmokers was significantly higher than smokers (p = 0.000) (Table 1).

*Gingival and Periodontal indices*

The mean of GI in nonsmokers and smokers were 2.9 ±0.25 and 1.5± 0.99, respectively. The mean of GI in nonsmokers was significantly higher than smokers (p=0). 

The mean of PI in nonsmokers and smokers were 2 and 2.90 ± 0.29, respectively. The mean of PI in nonsmokers was significantly lower than smokers (p=0) (Table 1). 

**Table 1:** Mean Mast Cell Density, Gingival Index, Periodontal Index and VEGF Expression Grades in Nonsmokers and Smokers

Factors	VEGF expression grading	No. of samples	Mast cell density (mean ± SD)	Gingival Index (mean ± SD)	Periodontal Index (mean ± SD)
Nonsmokers	Grade 0 Grade 1 Grade 2 Grade 3	1 5 7 17	5 7.8±4.08 11.42±7.04 9.05±5.72	3 2.8±0.44 3 2.94±0.24	2 2 2 2
Smokers	Grade 0 Grade 1 Grade 2 Grade 3	3 11 8 0	7.33±3.78 4.72±3.03 4.62±2.92 0	1.33±0.57 1.63±0.67 1.37±0.51 0	3 2.81±0.40 3 0

Mann-Whitney and ANOVA tests at the level of p < 0.05

*Packs × year assessment*

The minimum and maximum number of packs× years were 91 and 456, respectively. The mean of smoking duration was 256.6±102.3 packs × years. The mean of VEGF expression, mast cells density, gingival index and periodontal index were not significantly different in four groups of smokers (p=0.29, 0.12, 0.20 and 0.11, respectively) (Table 2).

**Table2:** Mean VEGF expression, Mast Cell Density, Gingival Index and Periodontal Index Based on Packs × Year Groups

Smokers (packs × year groups)	No. of cases	VEGF expression (mean ± SD)	Mast cell density (mean ± SD)	Gingival Index (mean ± SD)	Periodontal Index (mean ± SD)
Group 1	3	1.66± 0.33	7.00±1.52	1.33±0.08	2.66±0.16
Group 2	5	1.20±0.20	4.40±1.02	2.00±0.00	2.40±0.10
Group 3	8	1.12±0.29	5.12±1.07	1.31±0.20	2.75±0.13
Group 4	6	1.16±0.30	3.66±1.40	1.00±0.00	3.25±0.11

Duncan test at the level of p < 0.05

**Correlation between mast cell density and VEGF expression**

A strong, statistically significant correlation was found between VEGF expression and mast cell density (r= 0.27, df = 50, p = 0.000).

## Discussion

The present study indicated that mast cells density and VEGF expression in cigarette smokers is significantly less than nonsmokers in chronic periodontitis. 

Reduced inflammatory response and gingival bleeding in smokers compared to nonsmokers have been attributed to vascular density alteration. It has been shown that gingival vascular density is significantly decreased in smokers than nonsmokers (19- 20).

Reports have indicated that the number of small sized blood vessels in smokers is higher than nonsmokers (5-15).

The findings suggest that vasculature pattern of smokers is different from nonsmokers. The underlying mechanism by which cigarette smoking affects gingival tissue is still unclear. 

In an inflammatory condition, pro-angiogenic factors secreted by immune cells and newly formed vessels promote the passage of inflammatory cells to inflamed tissue (21).

Mast cells have a unique biologic feature because of involvement in chronic inflammation and even tumor development. Mast cells synthesize and release cytokines, growth factors and angiogenic compounds (22).

Chronic inflammation stimulates the proliferation of tissue mast cells population and recruitment of circulating precursors (23).

After activation, mast cells produce pro-angiogenic mediators such as VEGF and promote angiogenesis in inflammatory conditions (24).

VEGF has a mitogenic effect on arteries, veins and lymphatic derived endothelial cells (25).

VEGF introduces as a regulatory cytokine in both normal and abnormal angiogenesis. The results of present data indicate that gingivitis was more severe in nonsmokers than smokers. In sections obtained from nonsmoker and smokers, mast cells density and VEGF expression were significantly higher in nonsmokers than smokers. This finding is in consistent with the possible role of mast cells in development of inflammatory process in periodontium. Opposing results were obtained in relation to PI. The mean of PI in nonsmokers was significantly lower than smokers. The results are in agreement with some reports in the literature showing that mast cells density was less in chronic periodontitis than gingivitis or healthy samples (26-27).

Conflicting to these results, it has been reported that mast cell numbers are increasing in periodontitis (28-29).

The conflicting results come from different research designs. This study was planned to investigate the role of cigarette smoking on mast cell density and its association with gingivitis and periodontitis. 

 In a recent study, Huang et al. indicated a significant correlation between mast cell density and periodontitis severity. The authors tried to find an association between mast cell degranulation and periodontal disease in human (30).

Secretion of different immunologic related mediators including cytokines and prostaglandins is a key role of mast cells during the activation (31). 

 Reduced osteoclast activation and number of osteoclasts by blocking histamine uptake has been shown in mouse (32).

As histamine is a main product of mast cells, these cells can regulate bone destruction. In the present study, the mean of PI in nonsmokers was significantly lower than smokers. This is compatible with the adverse effect theory of cigarette smoking on periodontium. More research is necessary to explain the effect of smoking on mast cells role in periodontal tissue.

To investigate the relation between mast cells density and VEGF expression in chronic periodontitis of smokers, patients were classified into four groups based on the number of smoked cigarette. It was found that the mean of VEGF expression, mast cells density, gingival index and periodontal index were not significantly different in four groups of smokers. The number of smoked cigarette was not a determining factor on periodontal related disease, but cigarette smoking by itself had a harmful effect on tissue.

 Mast cells have multifactorial role in tissue new angiogenesis by producing different metalloproteases. Mast cells stimulate the migration and proliferation of endothelial cells in angiogenesis progression (33).On a mutual action, VEGF directly or indirectly recruits mast cells to tissues (34). 

The arising question is “what is the role of cigarette smoking on mast cell density and VEGF expression in chronic periodontitis?”

It is generally believed that vasoconstriction of gingival vessels is the main cause of suppressed inflammatory reaction in smokers. Decreased gingival bleeding is the clinical manifestation of this suppression. The vasoconstriction idea is not in agreement with other reports that showed smoking inhibits angiogenesis. Cigarette smoking inhibits normal process of angiogenesis and tissue repair (35). 

In the present study, we proposed a new theory for the mechanism of smoking on gingival vessels for the first time. We attributed decreased bleeding tendency in smokers to lower mast cell function. Mast cells are triggers of “angiogenic switch” and capable of developing new vessels by affecting other vasoactive molecules including VEGF.

To obtain more reliable results, male patients without any systemic diseases or taking medications entered the study in both nonsmoker and smoker groups. The patients were selected by their number of smoked cigarette. Patient selection in smoker group was not based on periodontal status. 

In the present study, the number of microvessels in correlation to mast cells density and VEGF expression was not investigated. Further research is needed to elucidate the correlation between microvessels and mast cells density in chronic periodontitis. Understanding the conducting role of mast cells on periodontal angiogenesis helps to develop therapeutic approaches by mast cell handling in chronic periodontitis.

Hence, it seems that smoking-cessation programs is still a critical policy for preventing oral cavity diseases and financial costs of periodontal related treatments.

## Conclusion

 Mast cell has a key role in angiogenesis by effecting angiogenic related molecules and has an influence on vascular pattern of chronic periodontitis in smokers. Suppressed number of mast cells in a smoker may lead to decreased number of new blood vessels formation.
